# Targeted Response to Elevated Bloodstream Infection Rates in Philadelphia Outpatient Hemodialysis Facilities, 2023

**DOI:** 10.1017/ash.2025.324

**Published:** 2025-09-24

**Authors:** Briana Bowen, Charlotte Galagher, Andrea Harmony, Jane Gould, Tiina Peritz

**Affiliations:** 1 Philadelphia Department of Public Health

## Abstract

**Background:** Hemodialysis effectively treats acute kidney injury and end-stage kidney disease. However, hemodialysis patients, requiring vascular access, have an increased risk of bloodstream infections (BSIs). Gaps in infection prevention and control (IPC) practices further increase this risk. Philadelphia outpatient hemodialysis facilities with elevated BSI rates were prioritized for Infection Control Assessment and Response visits (ICARs). **Methods:** We used National Healthcare Safety Network (NHSN) 2019-2023 dialysis data to analyze BSI rates in Philadelphia facilities using SASv9.4. We mapped facilities using ArcGIS, and analyzed relationships of facility size, community income levels, and area deprivation index (ADI) on the state and national level with the BSI rates.

We conducted ICARs from August 2024-January 2025 in facilities with BSI rates in the 75th percentile during quarter 4 (Q4) of 2023. ICARs included on-site observations of IPC practices using the Centers for Disease Control and Prevention (CDC) audit tools for dialysis and facility leadership interviews using the CDC dialysis ICAR tool. Feedback was provided verbally and through written summary reports. **Results:** NHSN data for 2023 included 227 BSIs, with 44 in Q4. Eleven facilities were in the 75th percentile for BSIs. BSI rates were higher in patients with central lines compared to those with fistulas and grafts (1.56 v 0.16), however, high facility catheter utilization rates were not associated with higher BSI rates (p=0.4923). Geospatial mapping revealed targeted facilities were spread across the city and not associated with income levels or high ADI. No correlations were identified between BSI rates and facility characteristics. ICARs were completed for 9/11 (81.8%) prioritized facilities. Frequently identified IPC gaps included: glove changes without hand hygiene, medication preparation in patient care areas, inadequate catheter hub scrub and dry times, and inconsistent station disinfection practices. Opportunities for improvement were identified in 15-38 (20.5-52.1%) of the 73 audit tool steps across facilities. Repeated high BSI rates did not predict more IPC gaps (p=0.3799), although more previous quarters with BSI rates in the 75th percentile were associated with a higher likelihood of having a high BSI rate in Q4 of 2023 (p=0.0041). **Conclusions:** Quarterly NHSN data can be used to prioritize facilities for ICARs and those visited had multiple IPC gaps identified. Many facilities in the 75th percentile had recurring high BSI rates over previous quarters, which could help better target facilities for IPC support. Surveillance is underway to determine if ICAR interventions will reduce the BSI rates in future quarters.

